# Speed and Accuracy of a Stepping-Over Motion in Older Women Under Dual-Task Conditions: A Cross-Sectional Study

**DOI:** 10.7759/cureus.101810

**Published:** 2026-01-18

**Authors:** Yusuke Maeda, Daisuke Sudo

**Affiliations:** 1 Physical Therapy, School of Health Sciences at Odawara, International University of Health and Welfare, Odawara, JPN; 2 Organization of Human Augmentation Research Center, National Institute of Advanced Industrial Science and Technology, Kashiwa, JPN

**Keywords:** dual task, elderly, fall prevention, motion capture, task switching

## Abstract

Background: Older women experience higher fall rates and are more susceptible to severe injuries than men. Therefore, fall-prevention programs should be specifically tailored for women.

Objective: This study aimed to examine the dual-task switching cost in older women and to clarify their association with the speed and accuracy of the crossing motion.

Material and methods: This study employed a cross-sectional design. A total of 20 older women (74.5 ± 1.7 years) and 20 younger women (21.5 ± 0.5 years) participated in this experiment. The crossing motion was measured using a 3D motion analysis system under four conditions: (1) lateral without a cognitive task, (2) lateral with a cognitive task, (3) backward without a cognitive task, and (4) backward with cognitive task.

Under each condition, participants were instructed to step over as quickly as possible in response to a visual cue. Each participant performed the motion 10 times per condition and was instructed to maintain consistent movements. The foot trajectory, variability of landed position, reaction time, and crossing time during the swing phase were calculated and compared between older and younger adults.

Results: Compared with the younger group, the older group showed a significantly longer reaction time during the cognitive task (p < 0.05). However, motion consistency and crossing time were comparable between the groups.

Conclusion: Shorten reaction time is essential for performing quick and accurate stepping movements, which are necessary for effective fall prevention.

## Introduction

In Japan's rapidly aging society, 'falls among the elderly' are not merely personal accidents but represent a serious social problem, leading to increased medical expenses, reduced quality of life (QOL), and a heavier care-giving burden. A central concern is the high rate of falls among older women. According to the Comprehensive Survey of Living Conditions conducted by the Ministry of Health, Labour and Welfare (MHLW) in 2019, 'fractures and falls' were the fourth most common cause of long-term care needs among the elderly (13.0%), following dementia, cerebrovascular disease (stroke), and age-related frailty. The proportion of individuals injured by falls, particularly those sustaining fractures, was markedly higher in women than in men. Previous studies have reported that women have a 2.2-fold higher fracture rate than men [1]. This can be attributed to factors such as accelerated osteoporosis and decrease in skeletal muscle mass [2,3]. Consequently, older women are more susceptible to falls and more likely to sustain serious injuries, including fractures, when falls occur. Proximal femoral fractures, among the most severe types of fractures, often require long-term hospitalization, surgery, and rehabilitation, substantially increasing medical expenses. Furthermore, even after fracture healing, many cases become dependent on care, permanently increasing the care-giving burden on families and society. Falls resulting in fractures are the primary cause in bedridden older adults. Therefore, preventing falls in older women has become a crucial medical and social challenge.

Aging is associated with numerous changes in the neuromuscular system, leading to a general decline in motor function [4,5]. The axon diameter of muscle spindles becomes narrower, reducing conduction velocity [6]. Declines in lower-limb muscle strength are particularly prominent, including reduced ability of the knee extensors to generate stable and submaximal force [7]. Such neuromuscular deterioration is a key factor in impaired postural control and is closely associated with increased risk of falls in older adults.

When posture becomes unstable during movement and a fall is imminent, individuals must react quickly and execute an accurate stepping action. However, older adults are thought to exhibit large movement variability, difficulty in performing intended movements, and reduced movement accuracy. Moreover, they often struggle to react quickly and generate the appropriate motor output, which may be reflected in reduced lower-limb joint moments during stepping [8]. Delayed reaction time is another major factor contributing to impaired rapid movement. It has been reported that the reaction time gradually increases with age [9]. It is hypothesized that older adults lose the ability to process relevant information compared to younger adults [10]. This prolonged information processing time becomes even more pronounced as tasks require more attention [11].

Because daily activities frequently involve cognitive activity, postural control during cognitive tasks is critically important for older adults [12]. The cognitive load on postural control has a significant impact on older adults. Pellecchia et al. reported that center-of-pressure (COP) sway in older adults was greater during an information-reduction task than during the normal condition [13]. Difficulty in appropriately dividing attention and concentration during postural control may increase the risk of falls and reduce confidence in movement, thereby interfering with daily activities [14]. Older adults may experience declines in both motor and cognitive function during dual-task conditions due to aging and neurological degeneration [15,16]. Given that poor dual-task performance in older adults is associated with a higher rate of falls, evaluating motor function during dual-tasking is crucial [17]. To investigate postural control in older adults during cognitive tasks, it is necessary to examine not only the time taken to complete cognitive task but also the switching cost, the time required to transition from a cognitive task to a motor task. The shifting task within the methods of measuring switching costs includes tasks that require judging a given number as odd/even or larger/smaller and is considered a critical component of executive function in cognitive tasks [14]. Previous research indicates that switching from a cognitive task to an upper-limb motor task is more difficult for older adults than for younger adults [18]. Furthermore, according to the Posture-First Hypothesis, older adults are predicted to prioritize postural control over cognitive abilities, at least in situations where a threat to balance (fall risk) exists, to prevent falls [19,20]. We believe that investigating switching costs and the decline in motor task performance under dual-task conditions in older women can provide critical insights into fall risk and contribute to the establishment of effective fall-prevention approaches. To assess these points, this study focused on the consistency and speed of stepping movements. In this study, motor consistency was defined as a high degree of coincidence of foot trajectories when the same movement was repeated multiple times. Variability in foot placement position was also considered a potential indicator of motor consistency. Regarding motion speed, we hypothesized that characteristics of speed delay would become evident by distinguishing the switching cost of movement speed from the time required for the movement itself. Therefore, the primary objective of this study was to examine movement performance under dual-task conditions by verifying foot trajectory coincidence and movement speed during stepping over an obstacle. The secondary objective was to evaluate motor consistency in greater detail by verifying the variability of foot placement positions. This study will yield valuable results that will contribute to reducing fall rates and improving activities of daily living and QOL in older women. We hypothesized that older women would experience prolonged switching costs and reduced motor consistency.

## Materials and methods

Participants

This cross-sectional study compared younger and older women to examine age-related effects on the accuracy of the stepping-over motion. Sample size was estimated using G*Power 3.1.9 software. Based on an analysis of variance with α = 0.05, sufficient power (1-β = 0.95), and a large effect size (0.25), a minimum of 18 participants per group was required. Considering the potential dropouts, 40 participants were recruited. The study included 20 healthy older women (mean age 74.5 ± 1.7 years; height 153.4 ± 4.1 cm; weight 51.1 ± 7.2 kg) and 20 healthy younger women (mean age 21.5 ± 0.5 years; height 159.3 ± 6.0 cm; weight 51.6 ± 5.4 kg). Older participants were recruited through a senior citizens’ staffing service in Odawara City. All participants were independent in their daily activities. Exclusion criteria included history of stroke, severe diabetes, dementia, visual or auditory impairments, and the need for walking aids. Participants received a written explanation of the experimental procedures, potential risks, and their right to refuse to participate in the study. All participants provided written informed consent to participate in the study. This study was approved by the Ethics Committee of the International University of Health and Welfare (2020-9792-5).

Experimental setup

A 3D analysis system (Vicon Motion System Ltd., UK) was used to quantitatively evaluate stepping-over motion accuracy based on foot trajectory consistency. Nine cameras and four force plates (AMTI Inc., USA) were synchronized. The sampling rates of the cameras and force plates were 100 and 1000 Hz, respectively. We defined as the anterior direction (anterior, +), X as the lateral direction (right, +), and Z as the vertical direction (upward, +). Reflective markers 14 mm in diameter were placed according to the Plug-in Gait marker set at 39 locations (see Appendix). The red light was set at eye level for the participants to use as a cue to start the step motion.

Cognitive task

To evaluate switching costs by measuring information-processing speed and the degree of cognitive-motor interference, we established cognitive tasks requiring appropriate judgment of presented numbers. These involved the random vocal presentation of numbers from one to nine at two-second intervals. The stimuli were presented in a female voice within a high-frequency range, which is easier for older adults to hear. The sound source’s position and volume were fixed, and all participants’ ability to perceive it was confirmed beforehand. Two alternating tasks were used: (1) judging whether the number was greater or less than five, and (2) determining whether it was even or odd. Participants were instructed to respond verbally as quickly as possible.

Procedure

The experimental procedure followed that of previous studies [21]. Participants crossed over the obstacles to the right and backward, with a height equal to 15% of each participant’s height. The obstacle was placed 10 cm from each participant’s feet. They crossed the obstacle with their right legs and stopped moving after landing. The dominant foot was defined as the side where the ball was kicked, and all participants were right-foot dominant. They were instructed not to look at their feet during motion. They were asked to swing their legs to follow the same trajectory and land their feet in the same place as much as possible in each trial. In addition, they were instructed to start their step motion and swing as quickly as possible when the red light was turned on.

Four conditions were set: right crossing without a cognitive task, right crossing with a cognitive task, backward crossing without a cognitive task, and backward crossing with a cognitive task. In all trials, the participants were instructed to step over as quickly as possible when the light turned red. After the examiner provided a cue to prepare the participants, the red light was initiated at an arbitrary time, with a delay ranging from one to five seconds. In trials with a cognitive task, a red light was initiated while the voice presentation was ongoing. If a participant tripped or stumbled on an obstacle, the trial was excluded. The participants practiced the motion several times for each condition.

Data analysis

Trajectory of the Right Heel Marker

**Figure 1:**
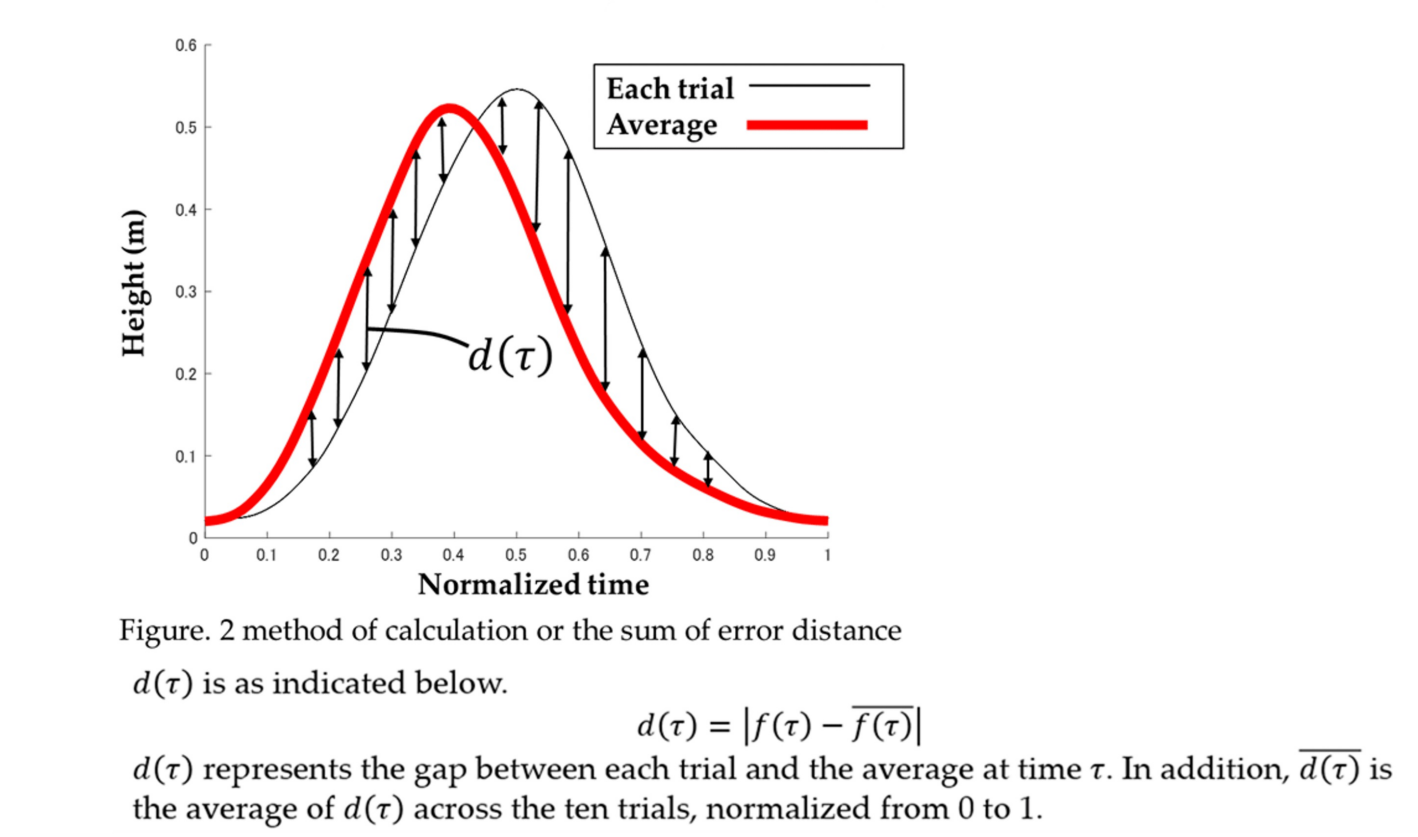
Method of calculation or the sum of error distance

Motion analysis software Visual 3D (C-motion, Inc., USA) was used to analyze the motion capture data. The data on the coordinates of the reflective markers during the swing phase were extracted. The start and end points of the data were defined as the moment when the heel marker moved 2 mm (heel off) and the force plate detected 10 N of foot contact (landing). Figure 1 shows the calculation method and the sum of the distance errors of the heel. Each data point was normalized from zero to one. To calculate the average trajectory of the 10 data points, curves were approximated using a cubic spline function and resampled at regular intervals. The gap between the average trajectory and each trial was summed and defined as the error distance. A large error distance indicated that the trajectories of the trials were not consistent.

Landing Position

To investigate the consistency of the landing positions, we recorded the coordinates of the right heel marker during landing. We calculated the SD of each coordinate of X and Y ten times and defined them as SD_X and SD_Y, respectively. The larger SD_X and SD_Y, the larger the error in the landing position in the anteroposterior and crosswise directions, respectively. This indicates a decrease in the motion consistency.

Reaction Time

Reaction time was defined as the interval between the onset of light and the initiation of the crossing motion.

Crossing Time

Crossing time was defined as the duration between the initiation of the crossing motion and landing.

Statistical Analysis

Statistical analyses were performed using SPSS (version 25.0; IBM, Inc., USA). The two-way repeated-measures Analysis of Variance (ANOVA) was used to examine main effects and interactions. When a significant main effect between the conditions was observed, Bonferroni-adjusted post-hoc tests were conducted. Statistical significance was defined as p < 0.05.

## Results

Tables 1 and 2 show the results for each outcome during the lateral and backward crossings, respectively. A simple effects analysis for lateral crossing indicated that the SD_X of young adults without cognitive task was significantly lower than that of older adults (p = 0.04). Additionally, the reaction time of younger adults with a cognitive task was significantly shorter than that of older adults (p = 0.01). Similarly, under the backward crossing, the reaction times of young adults, both with and without a cognitive task, were significantly shorter than those of older adults (p < 0.001). Both younger and older adults performed the cognitive tasks with near-perfect accuracy.

**Table 1:** The results of each outcome for lateral stepping-over motion

	Without cognitive task			With cognitive task			Main effect		Interaction age × cognitive task
							Age	Cognitive task	
Error distance (m)									
Young adults	0.027	±	0.004	0.029	±	0.016	F = 2.76	F = 0.65	F = 3.82
							p = 0.11	p = 0.43	p = 0.058
Older adults	0.035	±	0.019	0.032	±	0.018	ηp^2^ = 0.071	ηp^2^ = 0.018	ηp^2^ = 0.096
										SD_X									
Young adults	0.017	±	0.005	0.025	±	0.018	F = 5.58	F = 2.21	F = 2.03
							p = 0.023	p = 0.14	p = 0.16
Older adults	0.025	±	0.01	0.02	±	0.008	ηp^2^ = 0.13	ηp^2^ = 0.055	ηp^2^ = 0.051
										SD_Y									
Young adults	0.015	±	0.003	0.021	±	0.025	F = 1.83	F = 1.4	F = 0.21
							p =0.18	p =0.24	p =0.65
Older adults	0.021	±	0.006	0.034	±	0.063	ηp^2^ =0.46	ηp^2^ = 0.036	ηp^2^ = 0.005
										Reaction time (s)									
Young adults	0.31	±	0.09	0.43	±	0.1	F = 6.18	F = 73.6	F = 6.47
							p =0.017	p< 0.001	p =0.015
Older adults	0.33	±	0.12	0.54	±	0.1	ηp^2^ =0.14	ηp^2^ =0.66	ηp^2^ =0.145
										Crossing time (s)									
Young adults	0.75	±	0.11	0.65	±	0.11	F = 2.37	F = 0.82	F = 1.17
							p = 0.13	p = 0.37	p = 0.28
Older adults	0.8	±	0.21	0.8	±	0.43	ηp^2^ = 0.059	ηp^2^ =0.021	ηp^2^ =0.03

**Table 2:** The results of each outcome for backward stepping-over motion

	Without cognitive task			With cognitive task			Main effect		Interaction age × cognitive task
							Age	Cognitive task	
Error distance (m)									
Young adults	0.035	±	0.019	0.032	±	0.018	F = 0.64	F = 0.81	F = 0.47
							p = 0.43	p = 0.38	p = 0.52
Older adults	0.038	±	0.008	0.037	±	0.016	ηp^2^ = 0.017	ηp^2^ = 0.022	ηp^2^ = 0.012
										SD_X									
Young adults	0.025	±	0.018	0.024	±	0.02	F = 0.054	F = 1.21	F = 1.19
							p = 0.47	p = 0.28	p = 0.28
Older adults	0.031	±	0.011	0.025	±	0.009	ηp^2^ = 0.014	ηp^2^ = 0.031	ηp^2^ = 0.03
										SD_Y									
Young adults	0.035	±	0.03	0.033	±	0.035	F = 0.95	F = 3.91	F = 0.05
							p = 0.34	p = 0.055	p = 0.81
Older adults	0.028		0.009	0.022	±	0.008	ηp^2^ = 0.024	ηp^2^ = 0.093	ηp^2^ = 0.001
										Reaction time (s)									
Young adults	0.31	±	0.09	0.43	±	0.1	F = 7.87	F = 39.1	F = 0.037
							p = 0.008	p <0.001	p = 0.85
Older adults	0.41	±	0.11	0.52	±	0.14	ηp^2^ = 0.172	ηp^2^ = 0.51	ηp^2^ = 0.001
										Crossing time (s)									
Young adults	0.85	±	0.13	0.78	±	0.12	F = 0.21	F = 24.8	F = 0.013
							p = 0.64	p <0.001	p = 0.9
Older adults	0.87	±	0.16	0.79	±	0.12	ηp^2^ = 0.006	ηp^2^ = 0.40	ηp^2^ = 0.001

## Discussion

In this study, older women showed a significant prolonged reaction times, and a significant interaction was observed in lateral crossing, indicating that aging accelerates delay in reaction time. However, regarding movement accuracy, although slight reduction in movement consistency was observed in some measures, no prominent aged-related decline was found overall. Therefore, it is important to reduce the time from the signal onset to movement initiation for crossing actions during cognitive tasks aimed at preventing falls.

Consistent with our hypothesis, switching costs were increased in older women. In this study, a motor cue was presented while participants were performing a cognitive task, requiring rapid switching from a cognitive to motor task. In the context of fall prevention, this corresponds to situations in daily life where individuals must rapidly switch their attention to a motor task when faced with an external perturbation, such as stumbling or crossing an object, while engaging in cognitive activity. Switching costs are commonly reported to increase with age, which is consistent with the results of the present study [22,23]. Training involving cognitive or dual-task components has been suggested as an effective way for reducing switching costs. Studies have demonstrated a close link between cognitive and motor functions, such as research showing improved processing speed through instability-free weight resistance training, and studies reporting increased gait speed following home-based computerized cognitive training [24,25]. Balance training is hypothesized to promote cognitive improvement through cortical plasticity [26] and neurogenesis and is, therefore, considered an optimal method for reducing switching costs [27].

In contrast to our hypothesis, motor consistency did not differ significantly between age groups. In a similar experiment, older adults exhibited lower motor consistency than younger adults when performing a crossing task in a body-tilted position [21]. This indicates that under unstable conditions, compared with normal quiet standing, older adults exhibit greater variability in their movements and a larger discrepancy between their intended and actual actions. In the present experiment, no difference in motor consistency was observed because though a cognitive task was imposed, the movement was not performed under physically disadvantageous conditions. Alternatively, possible reasons for the lack of a clear difference in movement accuracy include a potential ceiling effect in the motor task and the possibility that participants had a high level of physical function because they were employable. In the present study, no age-related difference was observed in the speed of the crossing motion. Therefore, it is plausible that even when a decline in motor function is not detected, a delay in switching from a cognitive priority to a motor priority strategy caused by cognitive decline could increase the risk of falls. Therefore, we believe that fall-prevention training that involves cognitive or dual tasks is necessary to reduce switching costs in older adults.

This study has several limitations. First, participants demonstrated relatively high levels of motor function, which may not be representative of the motor abilities of their peers. Second, cognitive function was not directly assessed in older participants, preventing assessment of its potential influence. Third, the measurement of reaction time, from the visual stimulus during the cognitive task to the initiation of motor action, was interpreted as a decline in shifting task ability. However, because this process includes initiating movements based on visual perception, it may not reflect the ability to shift tasks. Although this study discussed brain function, no direct measurement of brain activity was conducted. Therefore, future studies should aim to quantify brain functions using functional MRI (fMRI) or functional near-infrared spectroscopy. For example, a dual-task performance effect is often inferred (or hypothesized) using fMRI. An eight-week combined cognitive and motor training program was observed to improve cognitive function and decrease prefrontal cortex activity [28]. Since such a decrease in activity in a specific region of the cortex is considered an improvement in efficiency, this suggests that dual-task training has a positive effect on the prefrontal cortex [29]. By simultaneously recording brain and motor functions during dual-task execution, the underlying mechanisms can be elucidated, which is expected to enable the development of effective fall-prevention programs.

## Conclusions

This study measured the speed and accuracy of crossing motions of older and younger women under single- and dual-task conditions using a 3D motion analysis system. The crossing motion was measured in two directions (lateral and backward) with and without concurrent cognitive task, resulting in four test conditions. Each participant performed 10 trials per condition and was instructed to execute the movements as quickly and consistently as possible. Compared with the younger group, older women exhibited significantly longer reaction time during dual-task performance, whereas no significant differences were observed in motion accuracy or overall crossing speed. Furthermore, because there was no significant difference in the speed of the crossing motion, these results suggest that reducing the reaction time could shorten the overall performance time, potentially leading to more effective stepping actions for fall prevention.

## Figures and Tables

**Figure 2:**
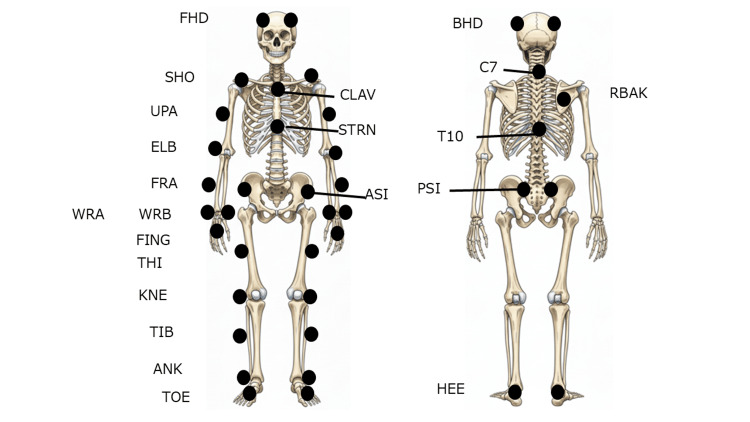
Marker placement FHD: Left and right skull of the frontal head, BHD: Left and right skull of the rear head, C7: 7th cervical spinous process, T10: 10th thoracic spinous process, CLAV: Suprasternal notch, STRN: Sternal xiphoid, SHO: Left and right acromioclavicular joint, ELB: Lateral epicondyle of the left and right humerus, WRA: Left and right radial styloid processes, WRB: Left and right ulnar styloid processes, FIN: Left and right second metacarpal head, UPA: Left and right center of humerus, FRA: Left and right center of ulna, ASI: Left and right anterior superior iliac spine, PSI: Left and right posterior superior iliac spine, THI: Left and right center of lateral thigh, TIB: Left and right center of lateral lower thigh, KNE: Medial joint space in left and right knee, ANK: Center of the lateral malleolus of the left and right ankles, TOE: Left and right second metatarsal head, HEE: Left and right calcaneus, RBAK: Right scapula
